# Early evolution of limb regeneration in tetrapods: evidence from a 300-million-year-old amphibian

**DOI:** 10.1098/rspb.2014.1550

**Published:** 2014-11-07

**Authors:** Nadia B. Fröbisch, Constanze Bickelmann, Florian Witzmann

**Affiliations:** Museum für Naturkunde, Leibniz-Institut für Evolutions- und Biodiversitätsforschung, Invalidenstrasse 43, 10115 Berlin, Germany

**Keywords:** fossil, amphibian, limb regeneration, Palaeozoic, Dissorophoidea, Temnospondyli

## Abstract

Salamanders are the only tetrapods capable of fully regenerating their limbs throughout their entire lives. Much data on the underlying molecular mechanisms of limb regeneration have been gathered in recent years allowing for new comparative studies between salamanders and other tetrapods that lack this unique regenerative potential. By contrast, the evolution of animal regeneration just recently shifted back into focus, despite being highly relevant for research designs aiming to unravel the factors allowing for limb regeneration. We show that the 300-million-year-old temnospondyl amphibian *Micromelerpeton*, a distant relative of modern amphibians, was already capable of regenerating its limbs. A number of exceptionally well-preserved specimens from fossil deposits show a unique pattern and combination of abnormalities in their limbs that is distinctive of irregular regenerative activity in modern salamanders and does not occur as variants of normal limb development. This demonstrates that the capacity to regenerate limbs is not a derived feature of modern salamanders, but may be an ancient feature of non-amniote tetrapods and possibly even shared by all bony fish. The finding provides a new framework for understanding the evolution of regenerative capacity of paired appendages in vertebrates in the search for conserved versus derived molecular mechanisms of limb regeneration.

## 1. Introduction

Regeneration of missing body parts occurs in most animal phyla, whereas regenerative capabilities vary extensively even between closely related taxa [1–3]. Much data have been gathered in recent years especially with a focus on the molecular and developmental mechanisms of regeneration and we may indeed be getting closer to a true understanding of its molecular basis [4]. By contrast, the evolution of regenerative capacity in animals and its ecological context has just recently shifted back into focus providing essential insights into the evolutionary history of regeneration [2,5]. Thereby studies concentrated on extant animal regeneration models to investigate the distribution of regenerative capacities in a phylogenetic framework and to assess which factors may have played a role in the loss or maintenance of it, such as direct selection, pleiotropy or phylogenetic inertia [2,3,6–8].

Among tetrapods, salamanders display by far the highest regenerative capacity that includes the eyes, heart, tails and entire limbs [1,9]. Therein, decades of research have been dedicated to the question of how it is possible for salamanders to repeatedly regenerate an entire limb in a matter of a few weeks and throughout their whole lifespan, while other tetrapods cannot [4,10,11]. The quest has undoubtedly been driven by the hope to eventually be able to induce human limbs to regenerate [11]. Most studies investigating limb regeneration have focused on the Mexican axolotl *Ambystoma mexicanum*, but limb regeneration has been demonstrated in a number of additional salamander taxa, including those that undergo direct development [5,12–15]. One of the most striking steps in the regeneration cascade is the de-differentiation of cells that had a specific, differentiated identity prior to the injury taking place, which re-enter the cell cycle to form a growth zone, the blastema [4,8,16]. The subsequent process of cell specification and pattern formation in the regenerating limb is not yet fully resolved. While grafting experiments and some molecular studies indicated that contrary to initial limb development, during regeneration the distal tip of the stump is specified first, followed by intercalary growth [17–19], more recent studies point towards a proximo-distal sequence of cell specification during regeneration, indicating that similar patterning modes may be used in development and regeneration [20,21]. The high regenerative capabilities of salamanders have classically been regarded as exceptional among tetrapods [3,5,8]. Among fish-like sarcopterygians (‘lobe-finned fish’), only lungfish are known to have a comparable capacity to regenerate their fore- and hind fins, including endoskeletal elements [22]. Contrary to salamander limb regeneration, however, the morphological and molecular aspects of lungfish fin regeneration have not been addressed in detail yet, but it is known that after the initial healing of a wound a blastema forms, which is overall comparable to the blastema initiating salamander limb regeneration [22].

Among amphibians, frogs display some regenerative capacity and can fully regenerate their limbs until the tadpole reaches metamorphic climax and similar molecular markers controlling certain aspects of the regeneration cascade have been found in premetamorphic frogs and salamanders [8,23,24]. As differentiation advances, the regenerative capacity of frogs gradually decreases and regenerative failure is correlated with an orderly reduction in the number of regenerated digits, inverse to the order of initial digit development [25] until regenerative capacity is lost in the adult animal with metamorphic climax [24]. Outside of sarcopterygians, a recent study showed that the basal actinopterygian *Polypterus* is capable of fully regenerating its pectoral fins at least until individuals reach reproductive age [26].

The question of which molecular and evolutionary differences between salamanders and other tetrapods are responsible for the high regenerative capacities of salamanders thus far remains largely unresolved. Many of the molecular mechanisms controlling regeneration of different tissues have been shown to be shared in animal regeneration [2,8]. However, limb regeneration is considered one of the most complex regenerative modes, and recent studies have identified a number of specific molecular markers that seem to be unique to salamander limb regeneration [24,27–30].

## 2. Material and methods

Specimens investigated for the study are housed at the Paläontologisches Museum Nierstein, Germany (SSN), the Museum für Naturkunde Berlin, Germany (MB), Institut für Geowissenschaften Johannes Gutenberg Universität Mainz (N), and the Staatliches Museum für Naturkunde Stuttgart, Germany (SMNS) under the collections numbers indicated. Specimens were investigated and photographed using a Leica MZ12 stereomicroscope and Leica DFC 420 camera set-up in combination with the Leica Application Suite Imaging Software. A thin layer of 70% ethanol was applied to fossils prior to photography to enhance visibility of bony elements.

## 3. Results and discussion

**Figure 1.**
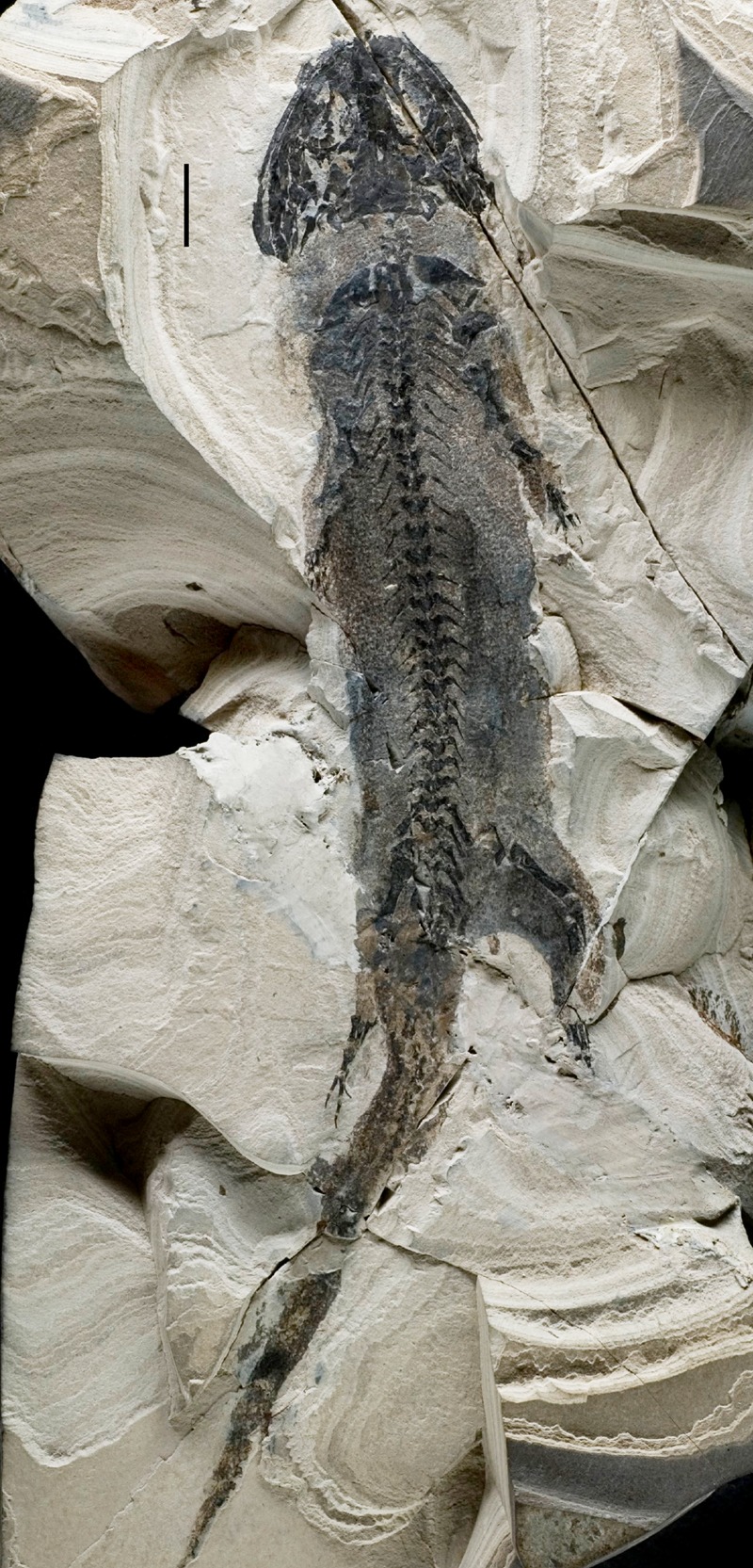
Whole specimen of *Micromelerpeton credneri*. Specimen MB.Am.1210 showing the exceptional quality of preservation of fossil amphibians from the fossil lake deposits of Lake Odernheim. Note the preservation of ‘skin shadow’, external gills, retinal pigments and scalation patterns. Scale bar equals 1 cm.

*Micromelerpeton crederni* represents a basal member of the dissorophoid clade within temnospondyl amphibians [31]. It is known from a large number of well-preserved specimens, which derive from *ca* 300 Ma old Upper Carboniferous to Lower Permian lake deposits in Central Europe. Anoxic bottom conditions in the lakes provided exceptional conditions for fossilization and specimens often preserve such detailed structures as external gills, stomach contents and scale patterns [32,33], which have provided exceptional insights into the anatomy, ecology and ontogeny of this taxon [34,35] (figure 1).

**Figure 2.**
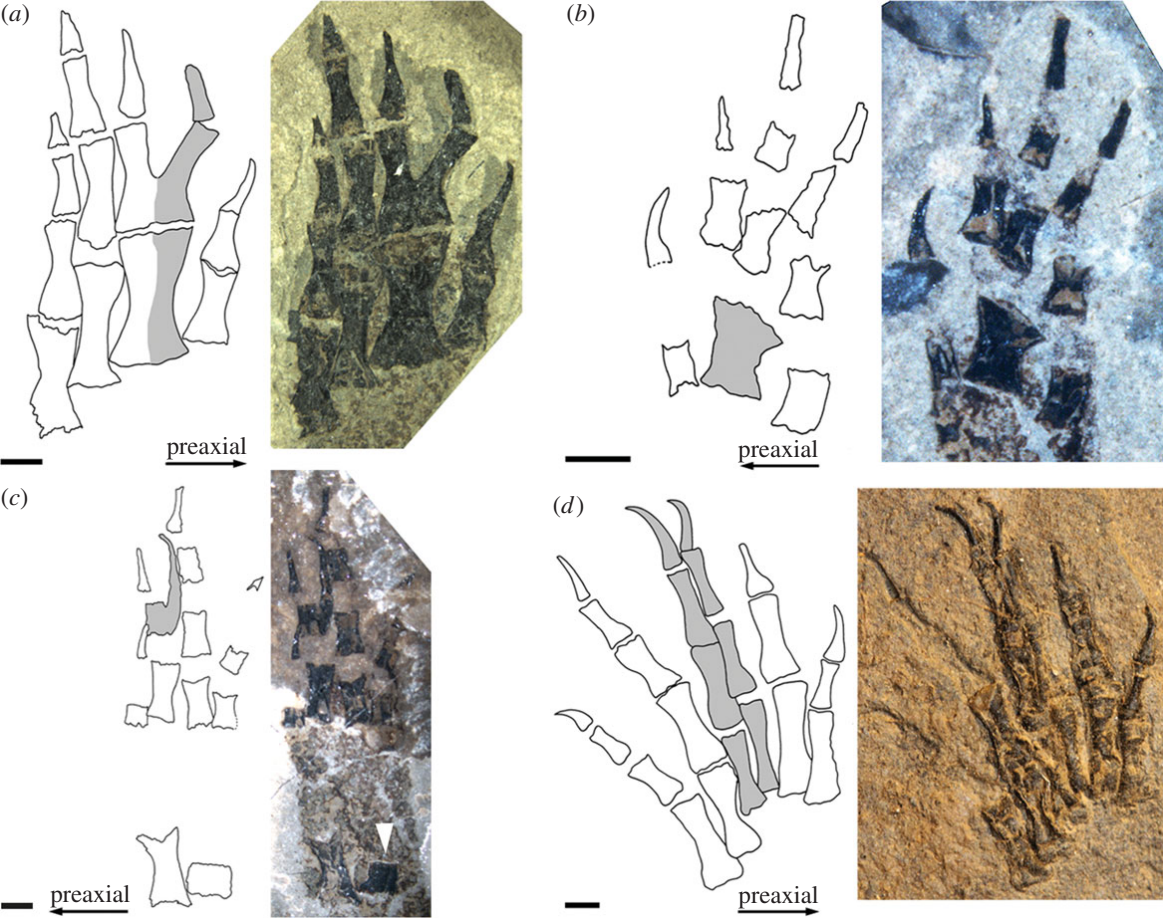
Examples for abnormalities caused by regeneration in *Micromelerpeton credneri.* Drawing (left) and photo (right) of some of the exemplar autopods displaying regeneration in *Micromelerpeton*. The normal condition is four digits in the hand with the phalangeal formula 2-2-3-3 and five digits in the foot with the phalangeal formula 2-2-3-4-3. (*a*) Right hand of specimen SSN 1102 showing enlarged metacarpal and proximal fusion of the first phalanges. (*b*) Left hand of specimen MB.Am. 1183 showing a fused metacarpal. (*c*) Left foot of specimen MB.Am. 1183 showing spur-like branching of the phalangeal element and an underdeveloped fibula (white arrow). (*d*) Left foot of specimen SSN GwK-34 showing a centrally positioned adventitious digit, note that both central digits are thinner than normal digits. Scale bar equals 1 mm. See also the electronic supplementary material figure S1.

Several specimens of *M. credneri* display limb abnormalities distinctive of irregular regenerative activity in modern salamanders. These include various degrees of fusion in the digits along the proximo-distal axis resulting in enlarged metapodial elements and distal bifurcations, predominantly in the preaxial region of the autopod (figure 2*a*–*c*). Moreover, some specimens show an addition of adventitious digits, both in the fore- and hindlimbs (figure 2*c,d*), whereas the adventitious digits are narrower than normal digits. A number of specimens display a reduction or an increase of phalangeal numbers in the regenerated digits (electronic supplementary material, figure S1 and table S1). In addition to the specimens that display the described limb abnormalities, a much larger number of specimens of *M. credneri* are known from various fossil localities, which document the normal autopod morphology and phalangeal count of 2-2-3-3 in the hand and 2-2-3-4-3 in the foot of *Micromelerpeton* [36]. Based on this, it can be deduced that in the variant patterns, additional digits occur preaxially, postaxially and centrally in *Micromelerpeton*, either in combination with a proximal fusion or without a clear association to a metapodial element. One specimen (MB.Am.1183, figure 2*c*) shows an underdeveloped fibula that is associated with a spur-like pathological phalanx in the foot of the same leg. Whether variant patterns were also present in the mesopodium (wrist and ankle) of *Micromelerpeton* remains unknown, because carpal and tarsal elements remained cartilaginous throughout most of the lifespan of this taxon and therefore did not fossilize. Moreover, although the frequency and diversity of variants in the limbs of *Micromelerpeton* are astonishing, it is not possible to reconstruct the real frequency of limb abnormalities within the population. This is because the fossil assemblages of Lake Odernheim are time averaged [37] and fossilization in general is influenced by a large number of random parameters, despite the exquisite preservational conditions and stratigraphic resolution (figure 1). Nonetheless, the abnormalities preserved in the ossified elements of the autopods of *Micromelerpeton* provide abundant insights into the number and combination of variant patterns in its limbs and are clearly associable with the specific variant patterns as produced by limb regeneration (see below; electronic supplementary material). They occur in both the autopods of fore- and hindlimbs of *Micromelerpeton* as would be expected in a random distribution of limb wounds and amputations in a natural environment caused by intra- and/or interspecific predation.

### (a) Abnormal limb regeneration

**Figure 3.**
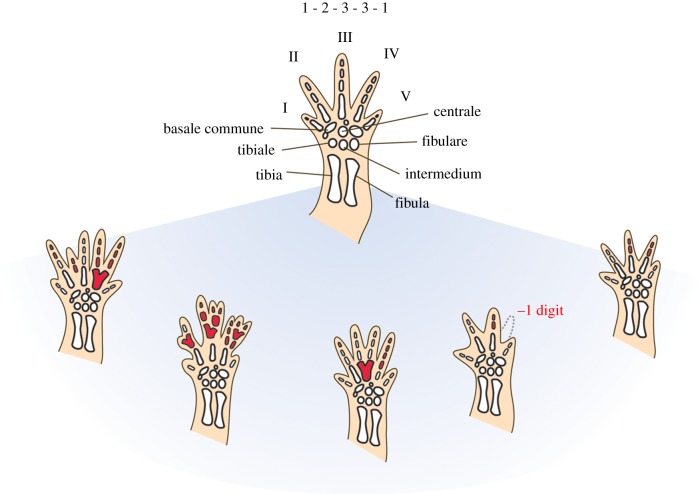
Range of patterns and combinations of abnormalities caused by regeneration. Regeneration causes a distinct pattern and combination of abnormalities in the limbs of salamanders including extra digits formed by branching or insertion of adventitious digits, missing digits caused by fusion or failure to regenerate, and an increase or reduction of phalangeal elements within digits. Different abnormalities within one limb or between different limbs of the same individual can occur. Hindlimbs of the salamander *Nothophthalmus viridescens* are depicted in this figure with the normal morphology of five digits and a phalangeal formula of 1-2-3-3-1 on top. Abnormal regions are highlighted in red. Data are based on the study of Stock & Bryant [38].

In modern salamanders, limb regeneration is a remarkably coordinated and smooth process and in most cases, regeneration produces structurally normal replacement limbs [12,38]. Once completed, it is difficult to differentiate the regenerate from an initially developed limb [4,10]. However, normal regeneration is dependent upon an orderly and accurate interaction between the different parts of the severed extremity to initiate the regeneration cascade and precisely replace the missing portions of the limb. It has been shown that a significant amount of tissue damage can lead to abnormalities and structure duplications [15], but even amputations without severe tissue damage can lead to abnormal regenerates with a variety of very distinctive abnormalities in the fore- and hindlimb depending on how the wound edges heal together [12,13,38]. These include extra digits formed either by branching or by insertion of adventitious digits, missing digits caused by fusion of adjacent digits and digital branches or failure to regenerate, and an increase or reduction of phalangeal elements within digits. Thereby a combination of different abnormalities within one limb or between limbs of the same individual are possible and frequent [12,38] (figure 3).

Variant patterns also occur during original limb development in salamanders. They seem to be particularly common in peripheral populations of some species [39–41] and are most commonly observed in the mesopodium (wrist and ankle) [39,41]. Notably, however, previous studies on extant salamanders have demonstrated experimentally that the typical pattern as well as the variant patterns produced by initial limb development vary significantly and qualitatively from pathologies associated with limb regeneration [12,13,38]. Variant patterns of limb development are characterized by interelement fusions of laterally adjacent cartilages in the postaxial part of the limb [13]. Contrary, pathologies associated with limb regeneration involve fusions along the proximo-distal axis and are frequently observed in the preaxial portion of the limb [13]. Moreover, pathologies associated with limb regeneration are most common in the autopods, while zeugopodial and stylopodial defects are rare and, if they occur, are accompanied by autopodial malformations [12]. Therein, repeated amputations seem to result in an increase in number of pathologies and their severity [12], reaching from a simple persistence of webbing between otherwise morphologically normal digits to severe deletions, fusions and abnormal ossifications in the autopodial skeleton [12,38]. Recently, it has been shown that malformations not only arise in the skeletal parts, but also in the limbs’ soft tissue with muscle abnormalities occurring in as many as 43% of regenerated forelimbs [42].

The fossil record shows that *Micromelerpteon* had alternative life-history strategies as known from modern salamanders including neotenic adults that retained an aquatic lifestyle and larval somatic features, as well as metamorphosed individuals [34,43]. However, a marked metamorphosis with a strong condensation of events comparable to that of lissamphibians likely only evolved within dissorophoid amphibians, while in most Palaeozoic temnospondyl the ontogenetic trajectory was much more homogeneous [44–46]. It is therefore difficult to identify a clear point in the ontogenetic trajectory at which the larval period ended, despite the excellent fossil record of *Micromelerpteon*. The assemblage of *Micromelerpeton* specimens relevant to this study includes larger larval as well as large, presumably adult individuals, but the material does not allow for an assessment when within the lifespan of an individual amputations and injuries took place, i.e. whether the regeneration of the respective limbs took place during the larval period and/or during adult life stages. Nonetheless, the pattern and combination of abnormalities in the limbs of the fossil amphibian *Micromelerpeton* are directly comparable to the variant morphological patterns in the limbs of adult extant salamanders, which have been demonstrated to be caused by limb regeneration, but do not occur as variants of normal limb development [12,13,38]. Like in abnormal regeneration of extant salamanders, variant patterns in *Micromelerpeton* consist of a number of fusions along the proximo-distal axis and abnormalities are predominantly located on the preaxial side of the autopods [13] (figure 2; electronic supplementary material, figure S1 and table S1). Additionally, specimen SSN GwK-34 displays six digits on the left foot. The adventitious digit was not produced by a distal branching of another element, but instead appears morphologically normal (figure 2*d*). It is, however, conspicuously narrower than normal digits, which is characteristic for adventitious digits in regenerated limbs of salamanders [38]. Specimen MB.Am 1183 shows an underdeveloped fibula associated with an abnormal spur-like protrusion on the first phalanx of digit II (figure 2*c*). This is similar to the reported zeugopodial abnormalities occurring in regenerated limbs of salamanders, which likewise always occur in association with autopodial malformations [12]. The most common variant pattern caused by abnormal regeneration in salamanders is an increase or decrease in the count of phalangeal numbers [38], which is also the most frequently observed abnormality in *Micromelerpteon* (electronic supplementary material, figure S1 and table S1). The distinctive parallels of the abnormalities in the limbs of *Micromelerpton* with those caused by abnormal limb regeneration in extant salamanders indicate that the temnospondyl *Micromelerpeton* was capable of regenerating its limbs.

### (b) Deep time evolution of appendage regeneration

**Figure 4.**
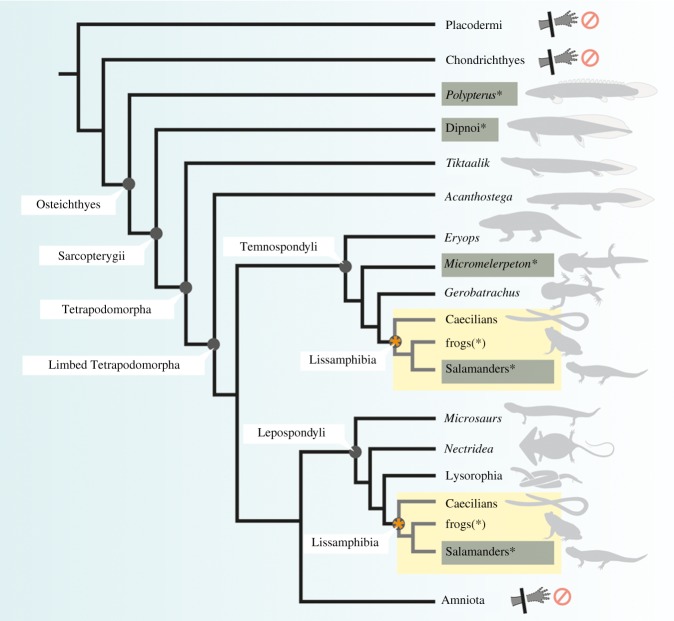
Regenerative capacity in vertebrates depicted in a phylogenetic framework. Taxa capable of limb regeneration are highlighted in grey with asterisk. Frogs only show regenerative capacity of the limbs until metamorphic climax (denoted by asterisks (*)). Taxa for which the lack of regenerative capacity in the limbs has been demonstrated are indicated with prohibition signs. Lissamphibians are highlighted in yellow in two alternative positions within the phylogeny marked by a star to represent alternative hypotheses for lissamphibian origins (see text). The phylogenetic distribution of regenerative capacity in paired appendages suggests the potential presence of plesiomorphic features of appendage regeneration in Osteichthyes.

The novel finding that the 300-million-year-old fossil amphibian *Micromelerpeton* was apparently capable of regenerating its limbs, for the first time enables a deep time perspective of the evolution of limb regeneration in vertebrates based on first-hand data from the fossil record (figure 4). Evidence for limb regeneration has previously been lacking from the fossil record, which is not surprising considering that usually only fully ossified skeletal parts are preserved in vertebrate fossils and in the vast majority of cases, fossils are incompletely preserved, missing individual skeletal elements or entire body parts due to local conditions at the time of preservation. This renders it almost impossible to unequivocally identify ongoing regeneration, since one cannot be sure if a limb or a part thereof has been lost due to incomplete fossil preservation or was indeed lost during the animal's lifetime. In the latter case, a still cartilaginous or poorly ossified regenerate would not be preserved in the fossil record.

The phylogenetic position of modern amphibians remains somewhat controversial. Most authors consider dissorophoid temnospondyls to be the closest Palaeozoic relatives of lissamphibians (the clade comprising modern frogs, salamanders and caecilians) [47,48], where *Micromelerpeton* represents the basal most member of the dissorophoid clade [31], while an alternative view places lissamphibians within lepospondyls [49] (figure 4). Among modern amphibians, frogs display considerable regenerative capacity as tadpoles, which however is lost in the adult animal [24,50]. Caecilians lack limbs and their capacity to regenerate other organs such as the tail or lenses as known from salamanders has thus far not been investigated experimentally (M. Wake 2013, personal communication). Among other vertebrates, regenerative capacity of paired appendages comparable to salamanders has been demonstrated only for lungfish [22] and the basal actinopterygian *Polypterus* [26], but seems to be lacking in chondrichthyians [51] and stem gnathostomes [52]. In teleost fishes, dermal fin rays can be regenerated to a certain degree, but teleosts lack the ability to regenerate bony skeletal parts of the fins [53,54]. In amniotes, limb regeneration is not possible [19] albeit a low regenerative capacity in the distal tips of digits of prenatal mice and chicks has been demonstrated [55]. Humans are also capable of regenerating fingertips whereas the capacity is highest in young children, but digit tip regeneration also occurs in adults [56].

The data suggest that *Micromelerpeton* was capable of regenerating its limbs and indicates that limb regeneration was likely an ancient capacity of the dissorophoid lineage leading towards modern amphibians that was retained in modern salamanders (figure 4). This is further supported by the still considerable regenerative capabilities of frogs until they reach metamorphic climax. In this scenario, the lack of regenerative capacity in adult frogs would represent a secondary loss, possibly correlated with the highly derived metamorphosis of anurans. Most other fossil tetrapod taxa from the same or a similar preservational settings (e.g. *Archegosaurus*, *Sclerocephalus*, certain microsaurian lepospondyls) are not preserved in the same number and/or detail as *Micromelerpeton* and despite an overall good fossil record often have only poorly preserved limbs. Therefore, no comparable evidence for regenerative capabilities could thus far be found in any other Palaeozoic candidate taxa. The only other dissorophoid clade with sufficient numbers and quality of preservation, the Branchiosauridae, does not show evidence of limb regeneration, although they share unique features in limb development with modern salamanders [57,58]. This, however, is not as surprising as it may seem at first, as it is well known from extant taxa, that a suite of evolutionary and ecological factors can influence the maintenance or loss of regeneration and the capacity can vary significantly between very closely related taxa [2,5,6,8]. Even within the salamander clade, some taxa cannot regenerate their limbs and their distribution does not follow any obvious phylogenetic pattern [8], though curiously, regenerative capacity seems to have been lost in those salamander taxa with strongly reduced limbs (sirenids, proteids and amphiumids). Dissorophoidea is a large and diverse clade with a 75 million-year-long evolutionary history [31,59], in which *Micromelerpeton* represents a basal member of the clade, as opposed to the derived position of branchiosaurids [31,60]. It is the nature of the fossil record that only a small fraction of the original diversity is preserved and an even smaller portion of that is represented in large enough numbers and detail to even allow for a search for possible signs of regeneration. Which factors may have influenced the evolutionary maintenance or loss of regenerative capacity in the dissorophoid lineage has to remain hypothetical. Ecology, pleiotropic effects, direct selection and phylogenetic inertia are some of the evolutionary drivers that are known to have played a likely role in other animal regeneration models versus their non-regenerating relatives [2,6,61,62]. Given what is known on the great diversity, ecology and ontogeny of dissorophoids [31,45,59,63], all these factors are likely to have played a role at one point or the other during the long evolutionary history of this clade.

When looking at an even broader evolutionary scale, the phylogenetic distribution of taxa that are able to fully regenerate their paired appendages could indicate that some fundamental aspects of fins and limb regeneration may even be an ancient feature for osteichthyians (figure 4). The fact that frogs and even amniotes retain some regenerative capacity in early phases of development may lend some support for this scenario and indicate that some of the molecular mechanisms allowing for regeneration of paired appendages are still present in modern tetrapods including amniotes, but repeatedly attained an ancillary role in the course of the long evolutionary history of the different osteichthyian lineages. On a greater developmental level, a number of widely shared features between various animal regeneration models have been recognized, which provide quite strong support for the homology of animal regeneration [1–3,7] and this may similarly be the case for molecular mechanisms involved in appendage regeneration on a higher ranking level. However, recent studies have identified a number of molecular markers involved in adult limb regeneration that seem to be unique to salamanders [29,30], including genes of the *Anterior gradient* (*Agr*) family [24] and the three finger protein Prod1 [27,28], which indicates that derived molecular mechanism play a central role in the great regenerative capacities of salamanders. Most authors agree that dissorophoid temnospondyls including *Micromelerpton* represent the stem lineage of modern amphibians and the capacity to regenerate limbs in *Micromelerpeton* may indicate that some of these derived molecular mechanisms could have evolved as early as the Lower Permian. The similarity between the variant patterns in the limbs of extant salamanders and *Micromelerpeton* caused by limb regeneration is striking and suggestive of shared molecular mechanisms that are still acting in modern salamanders as they did in their 300-million-year-old relative *Micromelerpeton*.

## Supplementary Material

Fröbisch et al._supplementary materials
