# Supplementary material for: Early evolution of limb regeneration in tetrapods: evidence from a 300-million-year-old amphibian
Source: Proc Biol Sci. 2014 Nov 7;281(1794):20141550. doi: 10.1098/rspb.2014.1550 (PMC4211449; doi:10.1098/rspb.2014.1550)
Supplement: Fröbisch et al._supplementary materials [file rspb20141550supp1.pdf]

Electronic Supplementary Materials for

Early Evolution of Limb Regeneration in Tetrapods - Evidence from a 300 Million year old Amphibian

Nadia B. Fröbisch, Constanze Bickelmann & Florian Witzmann

Supplementary Text - Anatomical Description of Abnormalities

Micromelerpeton is known from a large number of extraordinarily well-preserved specimens including many fully articulated skeletons often with preservation of soft parts like external gills, “skin shadows”, and retinal pigments (figure S1). They derive from fossil lake deposits in the Saar-Nahe Basin of southwestern Germany, which have already provided exceptional insights into the ontogeny of temnospondyl amphibians and their fossil ecosystem [1-4].

The normal phalangeal formula in *Micromelerpeton credneri* is 2-2-3-3 in the hand and 2-2-3-4-3 in the foot. Malz [5] and Boy [6] already discovered polydactyly (five digits in the manus) in three specimens of *Micromelerpeton*. Whereas Malz did not rule out that *Micromelerpeton* normally had five fingers (only very few specimens were known at that time), the five-fingered hands in *Micromelerpeton* were interpreted by Boy as “atavism”.

Specimen SSN 1102 has a well-preserved right hand with a phalangeal formula of 2-2-2-3-3 (figure 1*a*). Because digits II and III are partially fused, we assume that either digit II or III represents the additional digit (central polydactyly) rather than digit I. Metacarpals of digits II and III are fused completely, and the first phalangeal elements are fused proximally. The left hand also has five digits, however, due to compression, the phalangeal formula and a potential fusion of metacarpals are not identifiable.

A second specimen (SSN 1101) from the same locality has an additional digit in the left hand (figure S1*c*; see also [7], figures. 7, 8). In contrast to SSN 1102, one metacarpal and at least two phalanges of an extra digit were added postaxially, resulting in a phalangeal formula of 2-2-3-3-?2 [8]. The right hand is not preserved.

Specimen N 289 (also figured by Boy [6], figure. 20*d*) shows postaxial polydactyly in the left hand, with a presumable phalangeal formula of 2-?2-?3-?3-2 [6] (Figure S1*c*). The additional digit V is thinner and smaller than the normal ones.

In SSN 11066, the right hand is incompletely preserved with a presumable phalangeal formula of ?3-?3-?3-?3-2 (figure S1*g*). Not only has an additional digit developed postaxially, but also the number of phalanges in digits I, II, III and IV is increased. The left hand shows the normal condition of four fingers.

The left hand of specimen SSN OD 17-77 is well preserved and shows preaxial polydactyly (figure S1*a*). The phalangeal formula is 2-2-2-3-3. The right hand is not preserved.

In N 209, the left hand has an additional metacarpal (figure S1*d*). Because preservation is poor, the phalangeal formula cannot be determined. Only one phalangeal element per digit is preserved. The right hand has four normal digits.

Specimen SMNS 51316 shows polydactyly in both hands (figure S1*f*). Because of the considerably thinner and stouter shape of digit V in both hands, we hypothesize that the extra digits were added postaxially, resulting in the phalangeal formula 2-?2-?3-?3-2.

In specimen MB.Am.1183, the hands have four digits, but metacarpals II and III are fused in both hands (figure 1*b*). Furthermore, the phalangeal formula is reduced to 1-2-3-3.

Polydactyly also occurs in the pes of *Micromelerpeton* that normally has five digits with a normal phalangeal formula of 2-2-3-4-3. In the left foot of MB.Am.1183, only four metacarpals are present with the most proximal phalanx of digit II branching distally into a second phalangeal element (figure 1*c*).

Specimen SRK GwK 34 has six digits in the left foot (figure 1*d*). The phalangeal formula of 2-2-3-3-4-3 and the fact that digits III and IV are distinctly thinner suggests a duplication of digit III (central polydactyly). The right foot is incompletely preserved. Both hands show the normal condition of four digits.

Supplementary References

S1. Boy J.A. 2003 Paläoökologische Rekonstruktion von Wirbeltieren: Möglichkeiten und Grenzen. *Pal Z* **77**(1), 123-152.

S2. Fröbisch N.B., Carroll R.L., Schoch R.R. 2007 Limb ossification in the Paleozoic branchiosaurid *Apateon* (Temnospondyli) and the early evolution of preaxial dominance in tetrapod limb development. *Evol & Dev* **9**(1), 69-75.

S3. Schoch R.R. 2009 Life-cycle evolution as response to diverse lake habitats in Paleozoic amphibians. *Evolution* **63**(10), 2738-2749. (doi:10.1111/j.1558-5646.2009.00769.x).

S4. Witzmann F., Pfretzschner H.-U. 2003 Larval ontogeny of *Micromelerpeton credneri* (Temnospondyli, Dissorophoidea). *J Vert Paleo* **23**(4), 750-768.

S5. Malz H. 1970 Zur Deutung permischer Ur-Amphibien. *Natur Mus* **100**(10), 430-434.

S6. Boy J.A. 1972 Die Branchiosaurier (Amphibia) des saarpfaelzischen Rotliegenden (Perm, SW-Deutschland). *Abhandlungen des hessischen Landesamt fuer Bodenforschung* **65**, 6-137.

S7. Malz H. 1967 "Branchiosaurus", ein problematisches Ur-amphib aus dem Perm. *Natur Mus* **97**(10), 397-406.

S8. Boy J.A. 1971 Zur Problematik der Branchiosaurier (Amphibia, Karbon - Perm). *Pal Z* **45**(3/4), 107-119.

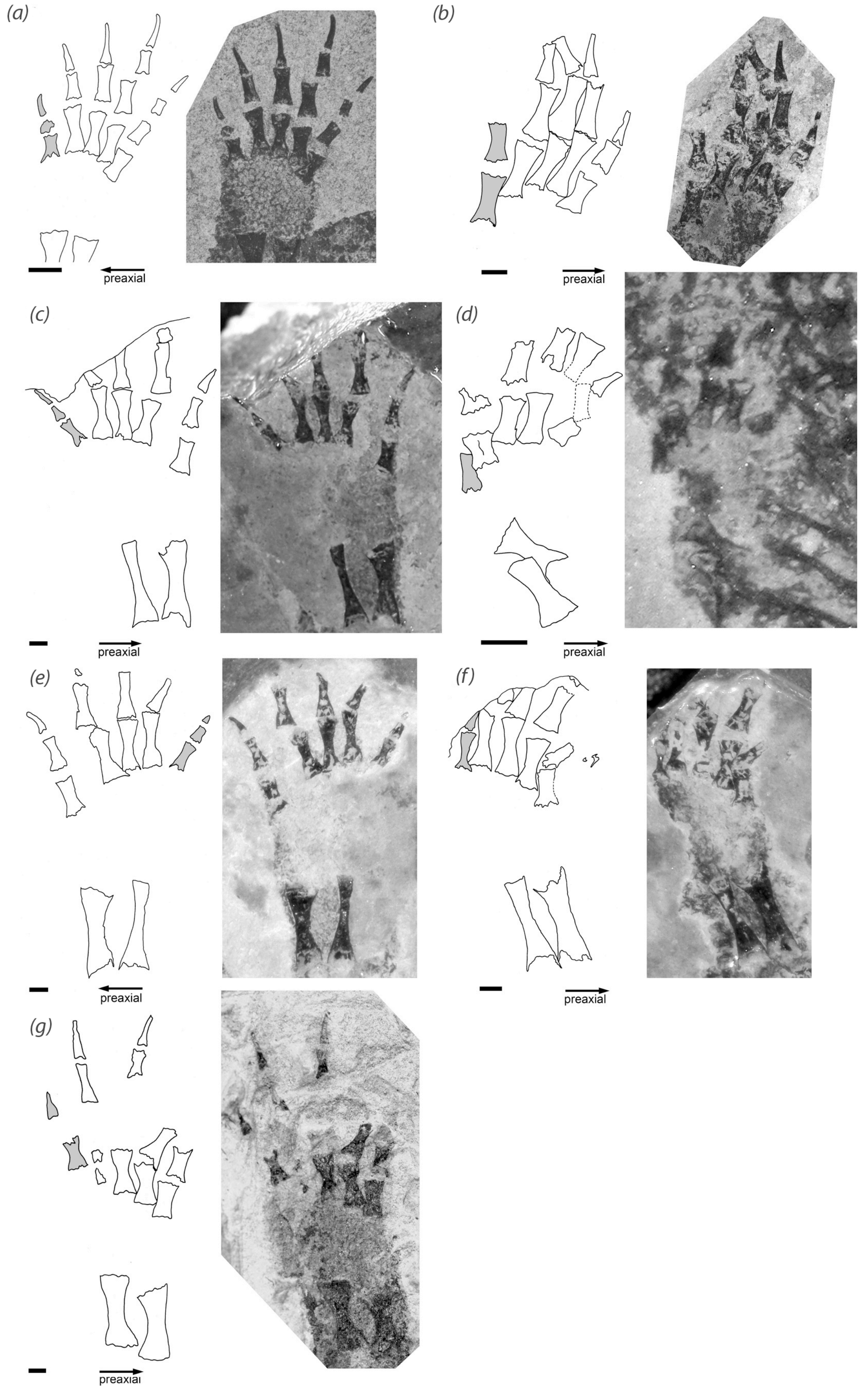

Figure S1.
Abnormalities caused by regeneration in *Micromelerpeton.* Regeneration causes a

distinct pattern and combination of abnormalities including adventitious digits, which can be added preaxially, postaxially, or centrally. (*a*) SSN OD 17-77 right manus with preaxial polydactyly and abnormal first phalanx. (*b*) SSN 1101 left manus with postaxial polydactyly. (*c*) N289 left manus with postaxial polydactyly. (*d*) N 209 left manus with postaxial polydactyly. (*e*) SMNS 51316 left manus with postaxial polydactyly. (*f*) SMNS 51316 right manus with postaxial polydactyly. (*g*) SSN 11066, right manus with postaxial polydactyly.

Table S1.
Depicting the range of specimens with abnormalities caused by irregular regeneration in *Micromelerpeton*.

**Collection No.**	**Phalangeal formula**	**Interpretation**	**Figure**
MB.Am.1183	hand: 1-2-3-3<br>foot: 2-2-3-4-3	fused metacarpals<br>branched digit II<br>underdeveloped fibula	2b<br>2c
N 209	-	additional metacarpal	S1d
N 289	2-?2-?3-?3-2	postaxial polydactyly	S1c
SSN OD17-77	2-2-2-3-3	preaxial polydactyly	S1a
SMNS 51316	2-?2-?3-?3-2	postaxial polydactyly	S1f
SSN GwK-34	2-2-3-3-4-3	central polydactyly	2d
SSN 1101	2-2-3-3-?2	postaxial polydactyly	S1b
SSN 1102	2-2-2-3-3	central polydactyly, fused metacarpals	2a
SSN 11066	?3-?3-?3-?3-2	postaxial polydactyly, additional phalanges	S1g
